# Geochemical dataset of the Danube Delta sediments

**DOI:** 10.1016/j.dib.2021.107529

**Published:** 2021-10-29

**Authors:** Laura Duţu, Dan Secrieru, Florin Duţu, Naliana Lupaşcu

**Affiliations:** The National Institute for Research and Development on Marine Geology and Geo-Ecology, GeoEcoMar, Romania

**Keywords:** St. George branch, Former meander, Sediment pollutants, Human intervention

## Abstract

We present geochemical analyses (major, such as CaCO_3_, TOC, Fe_2_O_3_, and minors such as MnO and TiO_2_) and trace elements, as well as some trace elements with genetic significance (Rb, Sr, Zr) or toxic and potentially affected by anthropic influences (Cu, pb, Zn, Cd, Cr, V, Ni, Co) of the superficial bed sediments of the St. George distributary which is the most sinuous and morphologically dynamic branch of the Danube River. Bed sediment samples were collected onboard of R.V. Istros, in June 2017. The dataset is useful to evaluate the distribution of the sediment along the fluvial channel, the present ecological state of the environment and, the effects of artificial intervention on the fluvial channel (the cutoff of the natural channels by navigational canals between 1984 and 1988). The data are presented as tabular format.

## Specifications Table

**Table:** 

Subject	Geochemistry
Specific subject area	Geochemical analyses of the superficial bed sediments
Type of data	Table Chart Figure
How data were acquired	Surface sediment samples were collected with a grab sampler “Van Veen” type. Sediments were oven dried and homogenized using a mortar grinder RM 200 (Retsch, Germany) and sieved with a 250-µm stainless steel sieve. The total organic carbon (TOC) and CaCO_3_ concentrations were determined in accordance with WakleyBlack titration method modified by Black [1] and Gaudette et al. [2], respectively. Fe_2_O_3_ (total), TiO_2_, Rb, Sr, Zr and V have been analyzed by X-ray fluorescence spectroscopy, using a VRA-30 sequential spectrometer, on compacted powders; Mn, Co, Ni, Cu, Zn, Cr and Pb have been determined by flame, AAS and Cd – by graphite furnace AAS on an ATI UNICAM 939E AA spectrometer, after digestion of the samples with boiling HNO_3_.
Data format	Raw Analysed
Parameters for data collection	Sediment samples were collected during a geological survey in June 2017 using a research vessel RV Istros, equipped with a grab sampler “Van Veen” type. The water discharge of the St. George distributary was 2170 m^3^.s^−1^ at the entrance in the study area (A1) (Fig. 1).
Description of data collection	Sediments were sampled in the Danube Delta (on St. George branch), along three cuttoff meander reaches (the Mahmudia, Dunavăţ de Sus and Dunavăţ de Jos meanders, named here as M1, M2 and M3 respectively), in June 2017 . Bed sediment samples (14 samples) were collected from the main channel of the St. George branch (Fig. 1). Two samples are located on the lateral canals (Perivolovca and Dunavăţ canals) and one sample on the Uzlina Lake.
Data source location	Institution: The National Institute for Research and Development on Marine Geology and Geo-Ecology – GeoEcoMar City: Bucharest Country: Romania The southern distributary of the Danube River, the St. George branch: Geographical borders of the survey area: UTM 35 N WGS84 Upstream bifurcation: 45^o^11′18.29″ N / 28^o^53′13.03″ E Distributary mouth: 44^o^52′45.33″ N / 29^o^37′04.26″ E
Data accessibility	Data are presented with the article

## Value of the Data

• Geochemistry of river sediments are controlled by the physical and chemical weathering of parent rocks, but also by factors such as climatic, hydrological, morphological and anthropogenic pressures in the basin.
• The presented data could be used by hydrologists, sedimentologists and geochemists for the research planning, sediment sampling, and specific analyses protocol.
• Evaluating post effects of the anthropogenic pressures in a natural environment can provide important comprehension of other similar cases worldwide.
• The data advance knowledge about actual ecological state of the Danube Delta sediments.

## 1 Data Description

**Fig. 1:**
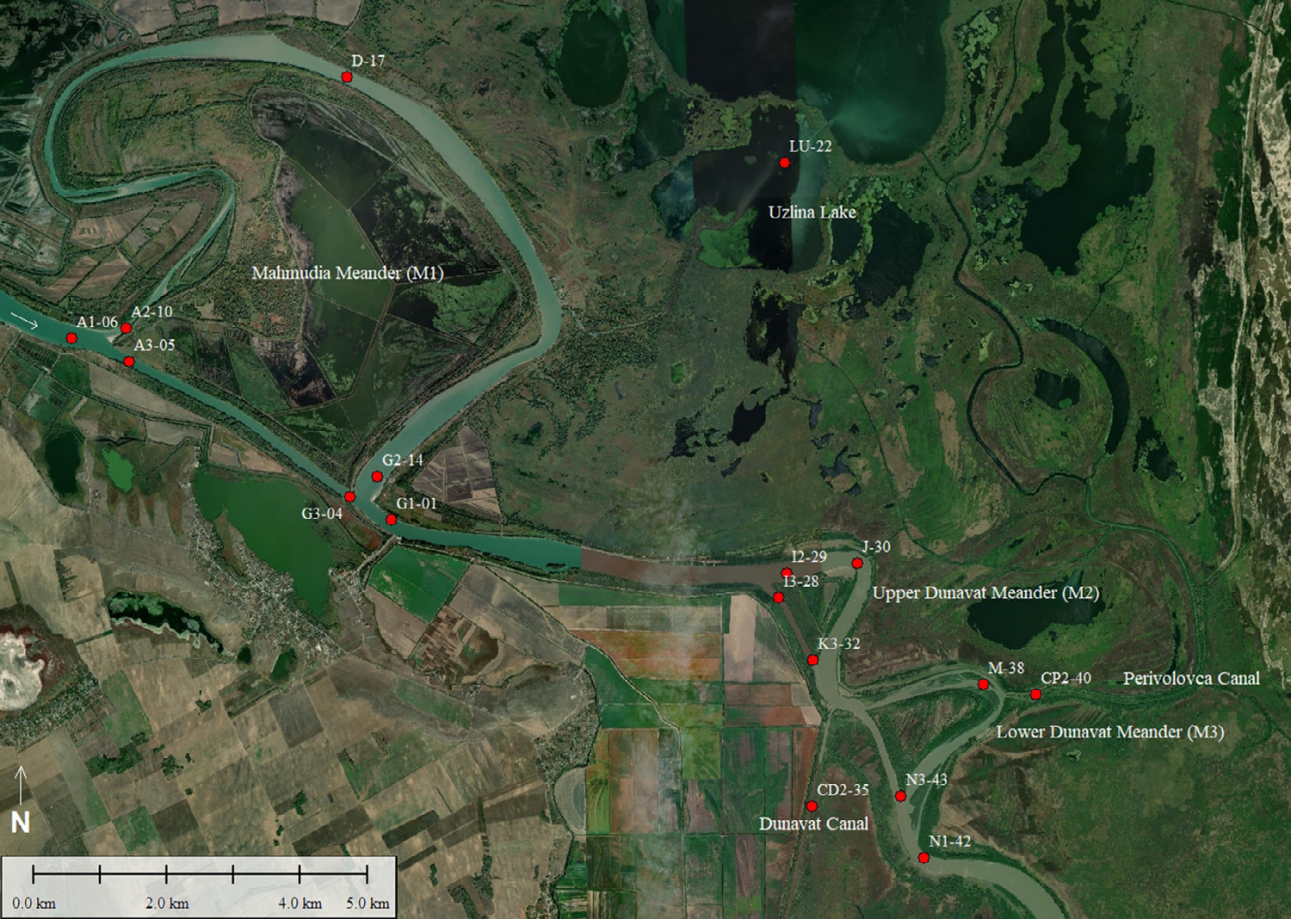
Location of the sediment sampled position. The map was obtained using Global Mapper 16 software.

This data presented consists of geochemical data acquired along the St. George branch in June 2017. The spatial distribution of the geochemical sampling stations is presented in Fig. 1. The sediments were sampled in medium to high water stage, at a daily discharge of 2170 m^3^.s^−1^ (measured at the entrance in the study reach, A1) (Fig. 1). Surface sediment samples were collected with a grab sampler from 17 locations distributed along the branch, on the main channel and former meanders (Mahmudia, Dunavăţ de Sus (Upper) and Dunavăţ de Jos (Lower)). Information on the sampling stations (coordinates, water depth and site characteristics are provided in Table 1.

**Table 1:** Location of the samples, site description and water depth.

Sample	Latitude	Longitude	Site Description	Depth
**G1-01**	45° 02′ 45.8962″ N	29° 11′ 24.4993″ E	Downstream confluence of M1	8.8
**G3-04**	45° 02′ 57.6957″ N	29° 10′ 56.4003″ E	Artificial canal at the confluence of M1	20.4
**A3-05**	45° 04′ 06.2048″ N	29° 08′ 28.2991″ E	Artificial canal at the bifurcation of M1	17.4
**A1-06**	45° 04′ 17.8985″ N	29° 07′ 49.5995″ E	Upstream bifurcation of M1	10.1
**A2-10**	45° 04′ 21.9034″ N	29° 08′ 26.6989″ E	Downstream bifurcation of M1	1.5
**G2-14**	45° 03′ 07.2065″ N	29° 11′ 15.9008″ E	Upstream confluence on M1	2.3
**D-17**	45° 06′ 21.0997″ N	29° 11′ 02.0997″ E	M1 natural course	4.5
**LU-22**	45° 05′ 33.5043″ N	29° 16′ 00.0003″ E	Uzlina Lake	1.0
**I3-28**	45° 02′ 03.1964″ N	29° 15′ 47.1994″ E	Downstream bifurcation on artificial canal of M2	4.4
**I2-29**	45° 02′ 14.9058″ N	29° 15′ 53.2002″ E	Downstream bifurcation on M2	9.0
**J-30**	45° 02′ 18.8989″ N	29° 16′ 41.6988″ E	Apex of M2	15.8
**K3-32**	45° 01′ 32.1961″ N	29° 16′ 09.3996″ E	Upstream confluence on artificial canal of M2	7.8
**CD2-35**	45° 00′ 21.5053″ N	29° 16′ 05.9015″ E	Dunavat Canal	5.6
**M-38**	45° 01′ 18.5980″ N	29° 18′ 05.3013″ E	M3 natural course	3.1
**CP2-40**	45° 01′ 13.0000″ N	29° 18′ 41.2000″ E	Perivolovca Canal	3.0
**N1-42**	44° 59′ 55.3972″ N	29° 17′ 21.5997″ E	Downstream confluence of M3	12.5
**N3-43**	45° 00′ 25.3967″ N	29° 17′ 06.7008″ E	Upstream confluence of M3 on artificial canal	21.8

Order 161/2006 [3] sets Romanian quality criteria for a number of chemical compounds, organic and inorganic, in sediments: these include a number of heavy metals analysed in sediment samples (Table 2).

**Table 2:** Quality criteria for heavy metals in sediments (Order 161/2006).

Metal	UM	Ord. 161/2006
Total chromium (Cr^3+^+Cr^6+^)	µg/g	100
Copper	µg/g	40
Lead	µg/g	85
Zinc	µg/g	150
Nickel	µg/g	35

The geochemical parameters analysed on bed sediments of the three studied meanders during the field campaign from June 2017 are shown in Table 3.

**Table 3:** Results of geochemical analyses on bed sediments of the three studied meanders during the field campaign from June 2017 to estimate the geochemical properties of bottom sediments and characterize the variation of major and trace elements.

Sample	CaCO_3_, %	TOC, %	Fe_2_O_3_, %	TiO_2_, %	Zr, µg/g	Sr, µg/g	Rb, µg/g	Zn, µg/g	Ni, µg/g	MnO, %	Cr, µg/g corr	V, µg/g	Co, µg/g	Pb, µg/g	Cu, µg/g	Cd, µg/g
G1-01	7.22	0.07	2.30	0.48	98	185	55	24.54	23.9	0.024	31.5	46	6.83	9.717	4.367	0.07
G3-04	8.23	0.04	2.80	0.59	112	186	53	20.83	18.1	0.021	30.4	44	4.64	8.762	3.86	0.071
A3-05	11.41	0.02	2.60	0.42	92	199	48	26.07	23.0	0.033	31.7	66	7.38	10.81	4.211	0.073
A1-06	7.66	0.05	2.44	0.51	94	189	51	18.81	17.7	0.019	28.6	43	3.88	9.019	2.934	0.056
A2-10	10.64	0.07	2.73	1.13	192	190	47	19.22	14.9	0.021	14.0	77	3.25	8.62	4.064	0.064
G2-14	10.16	0.95	6.09	0.98	272	196	109	103.40	50.7	0.107	89.7	114	12.60	24.2	44.84	0.296
D-17	9.67	1.15	7.54	1.01	187	185	134	151.10	73.9	0.111	127.0	144	16.28	38.18	69.80	0.427
LU-22	21.18	2.60	5.18	0.45	103	290	91	119.10	51.4	0.133	55.6	64	11.81	38.06	69.62	0.571
I3-28	9.14	0.01	3.17	0.93	117	176	37	16.12	14.0	0.019	19.2	89	3.31	7.529	3.00	0.048
I2-29	8.96	0.06	2.83	0.63	99	187	49	21.88	19.5	0.020	18.0	43	5.46	7.968	3.02	0.079
J-30	12.13	0.24	4.11	0.80	292	194	75	37.78	28.2	0.050	48.4	64	6.44	11.8	11.01	0.125
K3-32	13.20	0.72	6.01	1.02	285	183	104	73.82	51.8	0.090	75.2	88	12.37	22.52	26.37	0.182
CD2-35	12.13	0.53	5.34	0.93	347	210	106	74.98	42.9	0.069	57.7	110	11.24	16.68	28.75	0.281
M-38	10.20	0.06	2.65	0.71	143	203	54	23.47	18.8	0.015	19.4	69	4.68	8.368	4.21	0.079
CP2-40	10.52	0.86	5.61	0.95	316	190	103	96.26	46.2	0.085	70.7	99	10.87	20.44	41.81	0.325
N1-42	6.95	0.06	2.34	0.62	137	191	54	19.90	17.8	0.020	22.1	76	4.09	8.585	3.64	0.058
N3-43	11.38	0.72	5.84	0.83	244	189	105	68.63	51.5	0.075	59.9	104	11.59	20.35	27.37	0.177

The Table 4 show the descriptive statistics for the concentrations of the studied variables. Table 5 present the correlation matrix (Pearson) for the major constituents, minor constituents and heavy metals.

**Table 4:** Descriptive statistics for the concentrations of the studied variables of the collected sediment samples.

	CaCO_3_ %	TOC %	Fe_2_O_3_ %	TiO_2_ %	Zr µg/g	Sr µg/g	Rb µg/g	Zn µg/g	Ni µg/g	MnO %	Cr µg/g	V µg/g	Co µg/g	Pb µg/g	Cu µg/g	Cd µg/g
Mean	10.634	0.484	4.092	0.764	184.117	196.647	75	53.877	33.191	0.0536	47.002	78.823	8.042	15.976	20.757	0.175
Median	10.2	0.074	3.17	0.8	143	190	55	26.07	23.86	0.032	31.734	76	6.826	10.81	4.367	0.079
St. Dev.	3.256	0.669	1.712	0.228	89.646	25.340	29.952	42.544	18.131	0.0395	30.725	28.782	4.067	10.051	23.279	0.153
Minimum	6.95	0.014	2.3	0.42	92	176	37	16.12	13.98	0.0150	13.953	43	3.248	7.529	2.934	0.048
Maximum	21.18	2.602	7.54	1.13	347	290	134	151.1	73.92	0.132	126.957	144	16.28	38.18	69.8	0.571
C_v_, %	30.62	138.28	41.85	29.91	48.69	12.89	39.94	78.97	54.63	73.71	65.37	36.51	50.58	62.91	112.15	87.65
Count	17	17	17	17	17	17	17	17	17	17	17	17	17	17	17	17

**Table 5:** Matrix (Pearson) of linear correlation coefficients for the major constituents, minor constituents and heavy metals r_17; 0.05; 95_ = 0.482.

	CaCO3 %	TOC %	Fe2O3 %	TiO2 %	Zr µg/g	Sr µg/g	Rb µg/g	Zn µg/g	Ni µg/g	MnO %	Cr µg/g corr	V µg/g	Co µg/g	Pb µg/g	Cu µg/g	Cd µg/g
CaCO3, %	1															
TOC, %	0.817	1														
Fe2O3, %	0.434	0.689	1													
TiO2, %	-0.021	0.051	0.567	1												
Zr, µg/g	0.191	0.200	0.666	0.714	1											
Sr, µg/g	0.854	0.779	0.161	-0.359	-0.112	1										
Rb, µg/g	0.381	0.668	0.969	0.480	0.691	0.180	1									
Zn, µg/g	0.517	0.841	0.926	0.356	0.459	0.394	0.923	1								
Ni, µg/g	0.457	0.753	0.965	0.385	0.529	0.257	0.970	0.960	1							
MnO, %	0.675	0.906	0.907	0.303	0.489	0.506	0.889	0.956	0.931	1						
Cr, µg/g corr	0.262	0.610	0.938	0.445	0.542	0.068	0.942	0.922	0.951	0.857	1					
V, µg/g	0.120	0.378	0.823	0.725	0.622	-0.091	0.786	0.733	0.755	0.632	0.782	1				
Co, µg/g	0.458	0.725	0.939	0.333	0.531	0.261	0.956	0.939	0.985	0.920	0.935	0.728	1			
Pb, µg/g	0.637	0.917	0.858	0.220	0.290	0.528	0.841	0.964	0.924	0.955	0.849	0.613	0.893	1		
Cu, µg/g	0.605	0.914	0.870	0.272	0.362	0.524	0.858	0.984	0.915	0.958	0.851	0.656	0.886	0.981	1	
Cd, µg/g	0.707	0.950	0.788	0.170	0.334	0.669	0.787	0.937	0.838	0.932	0.744	0.540	0.821	0.945	0.975	1

**Fig. 2:**
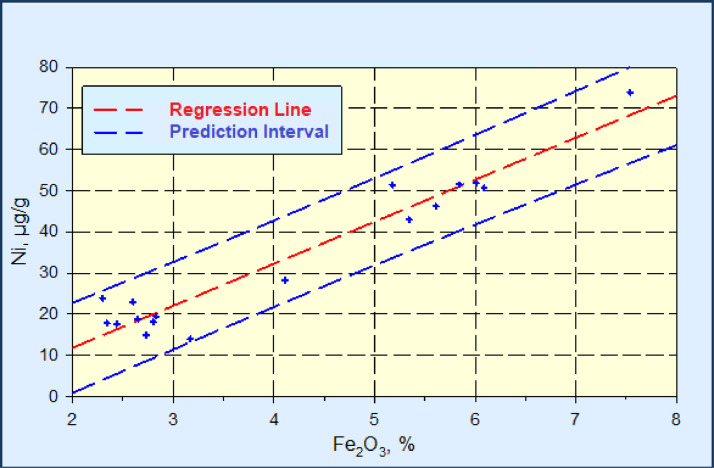
Linear regression diagram Fe_2_O_3_-Ni, with the prediction interval (C_Ni_ = 10.210*C_Fe2O3_ - 8.597, R^2^=0.930).

**Fig. 3:**
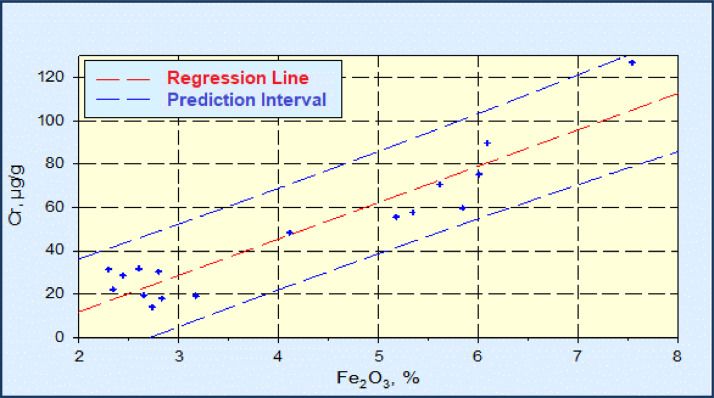
Linear regression diagram Fe_2_O_3_-Cr, with the prediction interval (C_Cr_=16.818*Fe_2_O_3_-21.831, r^2^=0.879).

**Fig. 4:**
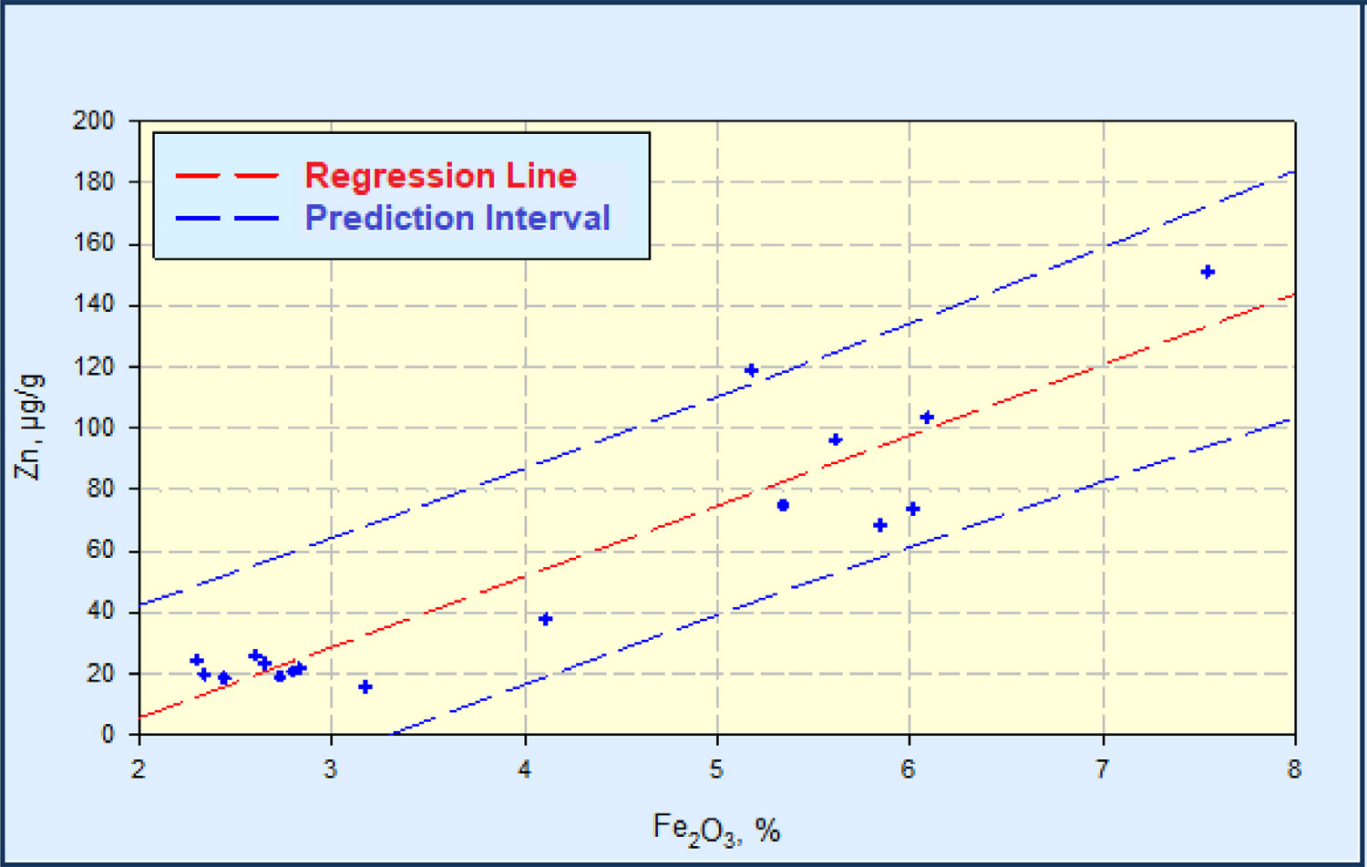
Linear regression diagram Fe_2_O_3_-Zn, with the prediction interval (C_Zn_=23.007*C_Fe2O3_-40.289, r^2^=0.858).

**Fig. 5:**
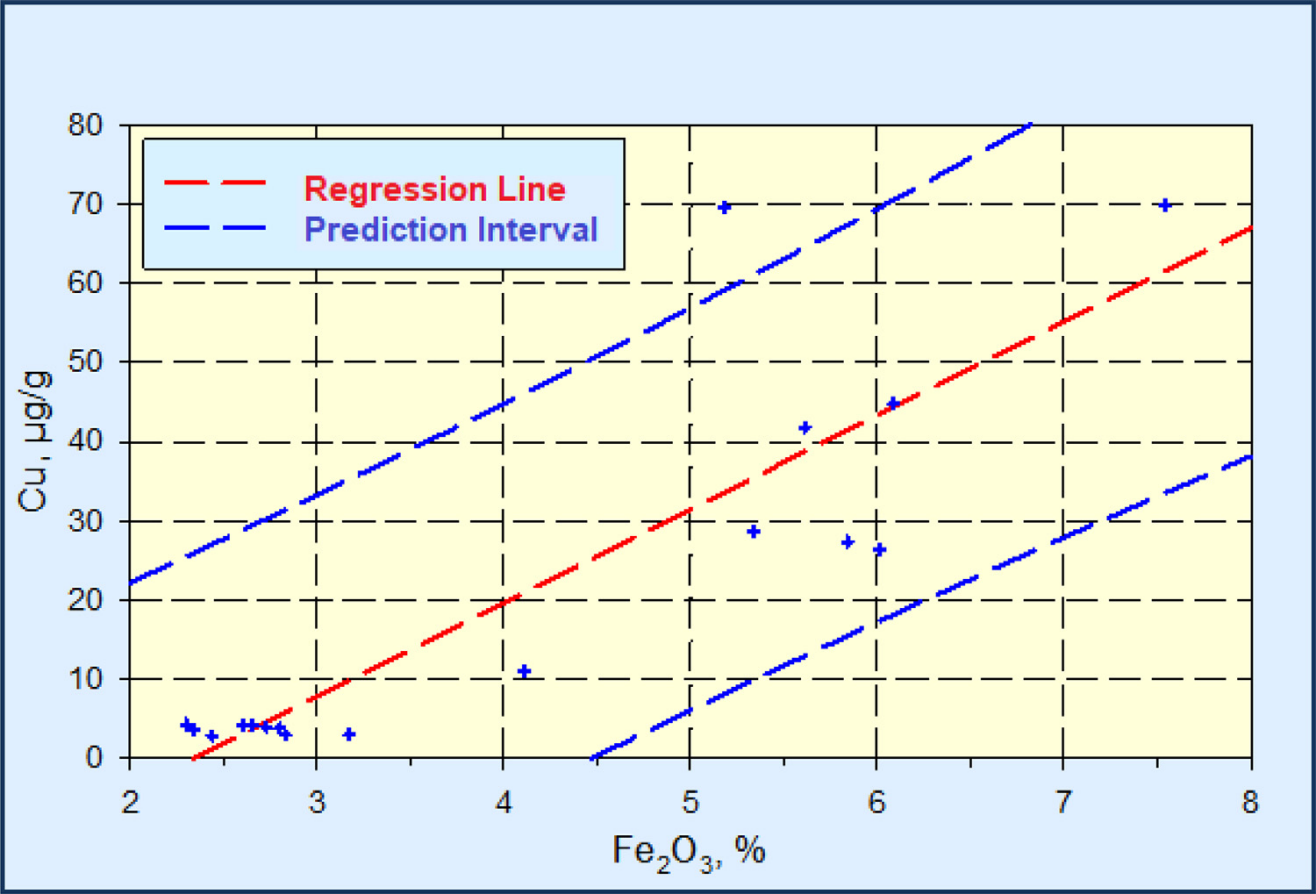
Linear regression diagram Fe_2_O_3_-Cu, with the prediction interval (C_Cu_=11.826*C_Fe2O3_-27.644, r^2^=0.757).

The linear regression analysis and the calculation of the prediction interval for the relationship Fe_2_O_3_ – Ni, Fe_2_O_3_ – Cr, Fe_2_O_3_ – Zn and Fe_2_O_3_ – Cu are represented in the Fig. 2, Fig. 3, Fig. 4, Fig. 5.

## 2 Experimental Design, Materials and Methods

Prior to geochemical analysis the sediment samples were oven dried (24–48 h/105°C), ground, and homogenized using a mortar grinder RM 200 (Retsch, Germany) and sieved with a 250-μm stainless steel sieve. The total organic carbon (TOC) and CaCO_3_ concentrations were determined in accordance with WakleyBlack titration method modified by [1] and [2], respectively. The concentrations of Fe_2_O_3_, TiO_2_, MnO, Zr, Cr, V, Zn, Cu, Ni, As, and Pb were measured by X-ray fluorescence spectroscopy using a VRA-30 sequential spectrometer, on compacted powders; Mn, Co, Ni, Cu, Zn, Cr and Pb have been determined by flame AAS and Cd – by graphite furnace AAS on an ATI UNICAM 939E AA spectrometer, after digestion of the samples with boiling HNO_3_. Accuracy and precision of AAS and XRF analyses were checked with several SRMs from US Geological Survey, NIST and IAEA.

## Ethics Statement

None.

## CRediT Author Statement

**Laura Duţu:** Conceptualization, Writing – review & editing; **Dan Secrieru:** Geochemical analysis, Writing – review & editing; **Florin Duţu:** Writing – review & editing, Data acquisition; **Naliana Lupaşcu:** Geochemical analysis.

## Declaration of Competing Interest

The authors declare that they have no known competing financial interests or personal relationships which have or could be perceived to have influenced the work reported in this article.
